# Conceptualising Lived Experience in Mental Health Research: Problems, Insights and Implications

**DOI:** 10.1111/1467-9566.70039

**Published:** 2025-04-30

**Authors:** Rajvinder Samra

**Affiliations:** ^1^ Department of Health, Wellbeing and Social Care The Open University Milton Keynes UK

**Keywords:** epistemic injustice, knowledge, lived experience, mental health, ontology

## Abstract

Across health research, drawing on accounts from people with lived experience is often promoted as a shift away from epistemic injustice wherein the knowledge of the marginalised is ignored/silenced. Paradoxically for people with mental distress who are given a diagnostic label, aspects of their accounts may actually be foregrounded to categorise them with mental illness/disorder. In doing this, their *sense of reality* (e.g., ontological experiences) can be judged to be significantly different from other people in addition to their epistemic knowledge and understanding. This points to issues of ontological differences between lived experiencers of various forms of mental distress that need deeper consideration in mental health research and practice, alongside the ongoing work on epistemic considerations. Experiencing serious mental distress can include a loss of trust in one’s sensory/perceptual signals which complicates how one makes sense of experience and knowledge. The complexities around these processes is at odds with the more straightforward ideas about testimonial accuracy and speaker credibility that underpin epistemic knowledge goals. This review outlines often‐overlooked issues related to ontological differences between people in the context of mental health and offers suggestions for the future.

## 1 Background

### 1.1 The Growing Use of ‘Lived Experience’ in Health Contexts

There are a range of perceived benefits of involving service users (the actual and intended recipients of a service) into healthcare education and training, and in service research and practice (Chambers and Hickey 2012; Roennfeldt and Byrne 2021). These groups of people can be referred to as those with ‘lived experience’ of particular health issues (Voronka 2016). In mental health contexts, professional roles for people with lived experience are expanding such that they can be employed or volunteer in services as lived experience advisors, peer workers or experts by experience, or may have a concurrent role as a healthcare provider (Roennfeldt and Byrne 2021). In research, lived experiencers can draw on the benefits of their expertise by collaborating and leading in research studies, contribute to research funding decisions and improve service design (Callard and Rose 2012). However, individuals’ internalisation of their lived experience of mental distress and the impact on their social identity is largely unknown and hypothesised to be complex (Gupta et al. 2023). The central focus on understanding lived experience in relation to the epistemic domain of knowledge and understanding, overlooks the ontological domain relating to an individual’s sense of reality which leaves important aspects of mental health lived experience unexplored. This article outlines some contentious issues in relation to lived experience in mental health contexts and offers suggestions for addressing them.

### 1.2 The ‘Lived Experience’ Concept

Although seemingly tautological in the English language, the term ‘lived experience’ has its roots in the German language (*Erlebnis*) through German philosophers (Burch 1990). The 19th century philosopher, Wilhelm Dilthey (1985), popularised the conceptual function of Erlebnis ‘that soon became so fashionable, designating a concept of value so self‐evident, that many European languages took it over as a loan word’ (Gadamer 1989, 61). In German, lived experience (*Erlebnis*) represents a more tacit and immediate experience in contrast to an experience from which one gains knowledge or learning from (*Erfahrung*) which Makkreel (1992) calls ‘ordinary experience’ (147). For a detailed examination of the development of lived experience within phenomenology see Burch (1990) or Casey (2023).

Dilthey described lived experience as a ‘reflexive or “self‐given” awareness’ (1985, 16) based on an immediate knowledge of reality. Ideas related to this system of experience and its conditions of possibility were notably developed in the German philosophical tradition but formulated quite differently by Husserl and Heidegger (Burch 1990). In the French language, lived experience has been used and developed by key thinkers associated with existentialism such as Simone de Beauvoir, Jean‐Paul Sartre (McBride 1981) and Maurice Merleau‐Ponty (Sadala and Adorno 2002). Merleau‐Ponty describes lived experience akin to a sensibility through our lived bodies as a type of situational and embodied form of knowing (Sadala and Adorno 2002).

More recently, van Manen (1990) provided an overview of the continental phenomenological movement and argued that lived experience gives rise to practical wisdom which can be useful for the human sciences. His classic text, ‘Researching Lived Experience: Human Science for an Action Sensitive Pedagogy’ (van Manen 1990), outlines the opportunities in operationalising and using lived experience material to develop a science dedicated to learning and understanding human experiences. Subsequently, specific methods inspired by the phenomenological tradition, such as interpretative phenomenological analysis (Smith 1996), demonstrated how to research lived experience in health in a detailed replicable manner. Methodological developments, including the increased use of interpretative phenomenological analysis, underscore how research using lived experiences has now become more commonplace in qualitative health studies (Eatough and Smith 2017).

### 1.3 Problems for Lived Experience in Mental Health Contexts

The way that lived experience narratives contribute to knowledge is not straightforward. For Eatough and Smith (2017), interpretative phenomenological analysis is interested in understanding the lifeworld (Lebenswelt) in which all knowledge is grounded—‘both our lived subjective knowledge and the objective knowledge of scientific abstraction’ (196, 197). van Manen (1990) sees phenomenological knowledge interactively, in that ‘we should refer questions of knowledge back to the lifeworld where knowledge speaks through our lived experiences’ (46). For van Manen (1990), phenomenological knowledge is dialogic, involves doing and is action sensitive because it is about ‘investigating experience as we live it’ (31).

Viewed in this light, lived experience has an intersubjective character involving relations between things. The lifeworld is both the source and the object of research (van Manen 1990) which complicates the relationship between lived experience and notions of scientific knowledge. Despite the complex interactional relationship, lived experience research in healthcare has become very closely and straightforwardly aligned to ideas of the epistemic (relating to the knowledge domain). For example, Bellingham et al. (2021) explore the knowledge of lived experience researchers during their mental health research training programme and argue that this represents a movement towards ‘epistemic justice doing’ (1588). The idea here is that epistemic justice is done by elevating the lived experience of some groups of people whose knowledge is systemically devalued due to social group prejudices such as being labelled with a mental illness/disorder. The recent focus on examining the lived experience of people in marginalised groups to promote epistemic goals raises the question of how this type of knowledge comes to be part of dominant scientific or objective knowledge bases, if at all.

In this article, I will demonstrate some of the outstanding problems and limitations of using lived experience narratives for pursuing mainly epistemic goals and I use the example of people with experiences of mental distress (‘lived experiencers’), some of whom may have been labelled with a mental illness/disorder. This work attempts to incorporate different paradigmatic approaches towards mental ill‐health with a view to encouraging those within them to interrogate their own assumptions about the limits of epistemic approaches. Very broadly, dominant psychological and psychiatric approaches offer ways of knowing that tends to promote or assert conceptions of normality and abnormality (N. Rose 2008) whether through social control or self‐regulation (N. Rose 1999). Such approaches can categorise mental distress based on ideas of normality, for example according the Diagnostic and Statistical Manual of Mental Disorders, 5th Edition (DSM‐5) (American Psychiatric Association 2013).

When deliberately drawing on these psychological/psychiatric (‘psy’) approaches, the present article will use terms such as ‘mental illness/disorder’ and any related diagnostic labels (e.g., depression or schizophrenia). There are many and varied criticisms of psy approaches, particularly throughout international Mad studies movements (e.g., Costa et al. 2012; Shimrat 1997; Beresford 2005; Sweeney et al. 2009). Mad studies scholars/activists and those from disability, queer, feminist and decolonial studies (and other fields) have demonstrated serious problems with the processes and practices associated with the psy disciplines (see LeFrançois et al. (2013) for more detailed exploration) that promote harm and injustice such as the inherent ‘othering’ intrinsic to diagnostic framing and categorisation. Mental distress might instead be communicating another way of knowing which is denied and pathologised as illness (Liegghio 2013). When deliberately drawing on and examining critical approaches, this work will use terms such as ‘mental distress’ to capture the range and continuum of mental ill‐health but also because such experiences do not need (or may not have) a label, and if a diagnostic label is given, it may be rejected by the lived experiencer of mental distress (Shimrat 1997). Although the concept of ‘lived experience’ in mental health is being used for this article, I accept that the term homogenises experiential knowledge and occludes diversity and difference (Voronka 2016). I am using it as a form of strategic essentialism in that I am acting ‘as if’ lived experience has a stable quality for practical or political purposes (see Gandhi 2015). I believe if we, as researchers and lived experiencers, explore ontological differences more explicitly as I am suggesting, we can develop more the appropriate terms and framing that we need to conceptualise lived experiences with greater granularity (in the context of the English language).

### 1.4 The Epistemic and Ontological in Mental Health

The recent focus on epistemic injustice in mental health (Kidd and Carel 2019; Kidd et al. 2023) tends to ignore or overlook the ontological domain (Samra 2023). The epistemic domain relates to knowledge, understanding and interpreting experience (M. Fricker 2007), whereas the ontological domain relates to the nature of reality (Bhaskar 2005) and one’s sense of reality and social realities (Jenkins 2020, 2023). The disproportionate focus on epistemic injustice in mental health research (particularly in the psy disciplines) produces pertinent problems for lived experiencers deemed to have a mental illness/disorder which centres on the mind/brain. Mental illness/disorder implicate one’s own epistemic architecture through its expression on thoughts, emotions and behaviours. For example, lived experiencers can have unusual reality (‘ontological’) experiences featuring contradictory, seemingly incomprehensible, impossible or factually inaccurate beliefs (Rootes‐Murdy et al. 2022). Their ontological experiences can be perceived by others (such as a psychiatrist) as not corresponding to dominant social or epistemic norms (based in facts or social facts) and they are judged as ontologically different. This categorisation can be harmful; for example, despite the stereotypes that people diagnosed with schizophrenia have irrational beliefs, studies also demonstrate that they might show no difference on tests of rationality (logic tasks) as people without the diagnosis (Revsbech et al. 2015). This judgement of one’s thoughts and opinions at a time of distress and suffering often serves to suppress their freedom of expression which can represent ‘ontological violence’ (Beaupert 2018, 769) because it can destroy their sense of self.

We need to understand different ontological experiences (Newbigging and Ridley 2018) beyond the focus on lived experiencers' epistemic contributions because this encourages foreclosure or limits our exploration to knowing, understanding or truth goals (epistemic). As is the case with all people, those in distress or not, lived experiencers can experience (or be encouraged to think they are) not knowing, not understanding and may experience incomprehensible, confusing and incompatible ideas that operate at the ontological level of their sense of reality. In pursuing epistemic goals, foreclosure on diverse ontological experiences can result in limited theorising about the ontological domain. For example, Bortolotti (2024) offers that ‘delusions are often a response to a puzzling reality’ or ‘a challenging reality’ (2) and both the speaker and interpreter should see them as meaningful because they have epistemic significance. I posit that we should also explore their ontological significance and meaning. Similarly, in Russell’s (2024) work combining philosophy and lived experience, the author also outlines the epistemic value of delusions as ‘interoceptive information’ (3) and ‘unconscious information’ (5). Both authors implicate the ontological domain without direct mention by drawing on the terms relating to internal bodily experiences and the unconscious which links to our first‐person sense of reality. Although there are epistemic benefits and value to delusions as argued by Bortolotti (2020), I am highlighting the language acrobatics that can avoid drawing explicitly on ontological terms and the consistent framing of ontological differences as informational and epistemic. I am suggesting that this is a limitation that we can overcome as researchers to more deeply explore what we appear to be referring to.

The focus on the knowledge and understanding of lived experiencers leaves these complex ontological experiences by the wayside, particularly anything that does not go on to become part of one’s epistemic contribution (knowledge‐related). Judgements about another's experiences have ontological significance as well as epistemic and, resultingly, researchers and practitioners in mental health contexts should consider ontological harm and justice. Problems inherent with the continual framing of pursuing distress narratives for epistemic goals in mental health research will now be described and suggestions for future research exploring ontological differences will be offered.

## 2 Mental Distress Lived Experience

### 2.1 Epistemic Injustice and Violence

Incorporating lived experience perspectives in health research appeals to a sense of social justice by promoting greater social inclusion. Historically overlooking, ignoring or silencing peoples’ perspectives if they do not share salient social positions of the dominant group (such as Whiteness, male gender or colonialist) maintains domination (Spivak 1994). Processes related to this type of exclusion have been variously labelled ‘epistemic injustice’ (M. Fricker 2007, 1), ‘epistemic violence’ (Spivak 1994, 76) and a type of ‘silencing’ (Hornsby and Langton 1998, 21). These types of exclusion have a long history in colonising cultures. For example, Dotson (2011) draws attention to the history of not recognising knowledge from women of colour in her article on tracking epistemic violence and practices of silencing.

Using lived experience in research can work to mitigate the effects of the systematic devaluation of particular experiences and knowledge based on the social location of the subject (e.g., their socially devalued race, gender or class). In her book, ‘Epistemic injustice: Power and the ethics of knowing’ (2007), the philosopher Miranda Fricker describes how ignoring the testimony of marginalised people due to social group devaluation ultimately undermines collective social understandings because it excludes their contribution to knowledge. M. Fricker (2007) uses the example of women whose accounts of sexual discrimination in the workplace were unfairly ignored or discredited on account of their gender rather than anything to do with the trustworthiness and credibility of their testimony (testimonial injustice). Fricker also outlines how denying people who experience sexual discrimination the collective resources for shared meaning‐making and understanding is an example of hermeneutical injustice. After extensive time and protest, the concept of ‘sexual harassment’ was eventually developed when individual testimonies were taken more seriously and shared meaning‐making took place. Subsequently, this concept has become part of dominant knowledge bases in many cultures through collective social understanding and sense‐making.

### 2.2 Social Understandings of Mental Distress

In M. Fricker’s work (2007), she describes that hermeneutical injustice is rooted in a lack of social interpretation for individual experiences which leaves one without the collective meaning‐making resources to understand particular phenomena. An individual can lack the necessary resources for self‐understanding due to having experiences that are occluded from wider examination. M. Fricker (2007) uses the case of Wendy Sanford as described in Susan Brownmiller’s account of the women’s liberation movement in the USA. Sanford was experiencing postpartum depression but did not realise what it was until she shared and discussed her experiences with a group of other women. Fricker describes this in terms such as ‘moment of truth’ (149) and ‘cognitive achievement’ (148) because her focus is on truth and knowing. Sanford’s realisation moves towards epistemic goals and Fricker situates this socially/collectively: ‘Here is a story of revelation concerning an experience of female depression, previously ill‐understood by the subject herself, because collectively ill‐understood.’ (149). Hermeneutical injustices are important to address, particularly in dismantling the isolation and stigma in particular lived experiences by supporting collective understandings of distress phenomena. M. Fricker (2007) demonstrates how mental ill‐health might be better understood if collectively metabolised using the example of a phenomena that others had experienced—postpartum depression. This skirts around a number of ongoing problems related to the idea of recognition in madness (see Rashed (2019) for a detailed discussion). For example, how do we affirm, identify or acknowledge something when we might not have encountered anything of its type *ever before*? Or how do we know our recognition is not a misrecognition (Rashed 2019)? There are additional complexities associated the harms of misrecognition for racialised minorities specifically as outlined by Fanon (see Morgan 2022). It is important to note here who tends to be excluded in ideas about sharing stories and collective meaning‐making; for example, there are particular capacities one needs to share their account and, additionally, some distress experiences can directly interfere with (or prohibit) sharing and communicating (Rashed 2019). Also, the idea in hermeneutical injustice that when self‐understanding is stagnated, it may need social/collective understanding situates one’s self‐understanding to some extent dependent on other social actors—which at once opens up the grounds of possibility but also limits them.

One of the problems with the power of collective understanding and shared sense‐making is that marginalised and racialised minorities are at a cultural and social disadvantage to being understood and this can still occur in well‐meaning research activities. For example, Mad studies scholars acknowledge that their own discipline suppresses and erases racialised people (Gorman and LeFrançois 2018). It is important to note how this relates to the ontological domain. There are individual and psychic (as well as social) elements to mental distress that may relate to one’s unique life history and cultural understanding of the world and one’s sense of reality. For example, in Black Skin, White Masks, Fanon ([1967] 2008) specifically uses ontological framing to describe how racism/colonialism causes harm to one’s mind (amongst many other harms): ‘The black man has no ontological resistance in the eyes of the white man’ (83) and as such, ‘the black is not a man’ (1). Not acknowledging someone as a person worthy of respect is a type of existential violence for the oppressed, which Fanon ([1967] 2008) argues leads to madness, demonstrating the importance of the ontological domain.

A lack of consideration of the ontological domain overlooks how people who are racially minoritised might see or experience reality differently or plurally, in part due to having different and/or multiple cultures or languages (as famously argued by Du Bois ([1903] 2017) in his conception of ‘double consciousness’). Similarly, Mills (1997) identifies the ontological struggle; one’s personhood being denied due to race has a name in Jamaican Creole, smadditizin’ which ‘refers to a deeper reality, a reality that without being pretentious—is properly called ontological’ (54). Irrespective of mental health, the racialised or colonised are considered ‘Sub‐persons with doubtful minds’ (Mills 1997, 61). Clearly, distress experiences are different for racialised minorities because of their social locations. For example, within the context of mental health, schizophrenia has been associated with White male genius and Black male dangerous criminality (Fernando 2018). This indicates differences in what is at stake for people in Black (and racially minoritised) groups in sharing their experiences whether in mental health research or services and might underscore differential willingness to engage in research or Mad activism. Without exploring differences between our ontological frames, we cannot assume that developing a shared interpretation is helpful or supportive to the lived experiencer because we do not know if the lived experiencer becomes further (e.g., racially or culturally) marginalised in these processes (Moodley et al. 2018).

## 3 Problems Inherent in Categorising Experiences

### 3.1 Psychiatrization

M. Fricker (2007) demonstrates how the testimony and accounts of marginalised people should be more fairly considered to promote social justice. Ethically, this also works to limit their continued societal exclusion which is part of the ongoing everyday injustice they face. However, there are complications and challenges related to extrapolating this line of argument to the inclusion of lived experiencers. Taking the example of a person with a diagnosis of mental illness/disorder, some (selective) aspects of their own accounts or testimony can be examined by a mental health professional to judge that they think or act in a manner that maps onto an a priori mental illness/disorder such as those outlined in the DSM‐5 (American Psychiatric Association 2013). People with mental illness/disorder are categorised as mentally different in a downwards comparison by a socially sanctioned professional (Foucault 2006; Newbigging and Ridley 2018). The psychiatric system marks them as ontologically different (Kecmanović 1983). At this juncture, this categorisation has ontological aspects (what constitutes reality and reality‐based assumptions) and epistemic considerations (relating to truth, accuracy and knowledge). Significantly, the professional categorisation of mental illness/disorder is in effect an ontological difference based on one’s sense of reality and how it differs from others (Castel et al. 1979; Kecmanović 1983). Therefore, psychiatry, in and of itself, can inflict damage through psychiatrisation because an individual’s experiences and expressions of distress are identified as mental illness/disorder (Kecmanović 1983; Castel et al. 1979).

A conventional diagnostic approach represents an a priori theoretical framework for unusual or uncommon experiences into conceptual categories such as those in the DSM‐5 (American Psychiatric Association 2013). There are many possible problems with this categorisation. For example, this categorisation could be inaccurate (neurodiversity instead of mental illness/disorder; Watts 2023), unjust because of discrimination and bias (racist overcategorisation of schizophrenia for Black individuals; Fernando 2018), or invalid because it is not deemed to be real (a person diagnosed with a ‘personality disorder’ who does not see it to be a real entity because it does not represent any reliable or valid underlying construct; Hartley et al. 2022). All three examples are potentially harmful but the first two (inaccurate diagnoses and discriminatory practices) relate to processes of knowledge‐related failures/errors within the context of the psy disciplines, whereas the final example is an ontological difference between the lived experiencer and the diagnostician. The person categorised with personality disorder in the final example is being labelled with something they do not consider to be (ontologically) real, so of course it is not (epistemically) true. We know less about the ontological harm or ontological violence that is enacted in the third example by our focus on epistemic harm. For example, the harms of the borderline personality disorder diagnosis (BPD, also known as emotionally unstable personality disorder EUPD) are often focused on in epistemic injustice terms (Watts 2024). However there have been calls in the psy disciplines to abandon the concept of BPD because it has no scientific basis (Mulder and Tyrer 2023). Resultingly, there is the potential for ontological harm because of the lack of evidence within the psy disciplines that it even exists as a construct.

In Mad studies, the types of debates emanating from the third example are commonplace, in that mental illness/disorder are not real constructs but research does not necessarily locate these arguments with reference to the ontological domain explicitly. For example, pioneers of Mad studies and disability activism movements in the UK, Peter Beresford and Diana Rose make many powerful arguments in their article on how paradigm differences create problems for knowledge in patient–public involvement endeavours (D. Rose and Beresford 2024). They identify a key problem is the ‘underlying assumptions of conventional researchers’ which differ from the ‘assumptions and aims of survivors’ (1) whilst not situating this as ontological, in fact they state: ‘That the assumptions take the form they do is a matter of epistemology’ (6). They criticise both psychiatry and survivor movements as having limited theory around contextualising the individual and state the need to ‘increase the bandwidth of what it means to be human’ (10). I would locate these lacks as an underdevelopment of theorising and researching around the ontological domain in UK Mad studies because of the focus on Mad knowledge (D. Rose 2023). It should be noted that a number of Canadian Mad studies scholars demonstrate a path forward by theorising directly about the ontological which might be inspired by explicit consideration of indigenous, racially minoritised and anticolonial worldviews and the promotion of equal rights in research (LeFrançois and Voronka 2022; LeFrançois et al. 2013). Helpfully, Voronka (2016) refers to ‘variant epistemological and ontological frames’ (190) between disciplinary approaches which might offer paradigm‐fluid language going forward.

### 3.2 Ontological Differences and Not Knowing

Another issue related to the domain of mental illness/disorder is that the lived experiencer is not necessarily a knower in conventional epistemic terms. There are important issues to address relating to one’s capacity to know, understand and make decisions. A systematic review of the prevalence of patients in psychiatric settings who were categorised as lacking capacity was 45% across 35 international studies (Lepping et al. 2015). As an example, in England and Wales, the Mental Capacity Act (2005) outlines that a person lacks capacity if they are ‘unable to make a decision for himself in relation to the matter because of an impairment of, or a disturbance in the functioning of, the mind or brain’ (Section 2). Additionally, it needs to be deemed the decision cannot be made because the person is not to be able to understand, retain, weigh up relevant information or communicate their decision (Section 3). Being assessed under this Act relates to making a specific decision but the categorisation of lacking capacity in this decision can also have undesirable consequences for lived experiencers (Series, 2025). Although the Mental Capacity Act can be considered empowering and protective, Series (2025) outlines: ‘Mental capacity law is also stigmatising, but the stigma is different: the paternalistic stigma of protecting vulnerable incompetent populations’ (9). There might be potentially destabilising effects, because of this stigma, on a lived experiencer’s perceptions about their broader knowledge, understanding or decision‐making that might accompany an assessment of lacking capacity in the past for particular decisions. This highlights particular vulnerabilities and sensitivities about knowledge and understanding for people who have been deemed to lack capacity in the past which warrants deeper consideration in mental health research.

n line with this, a tension within epistemic injustice for people with mental illness/disorder is the continued focus on ideas about the ‘knower’ or ‘epistemic agent’ (Kidd et al. 2023, 504). A number of mental illness/disorders can come with beliefs that other people judge to be false, unproven, inaccurate or disproportionate (Rootes‐Murdy et al. 2022), which makes it confusing to centre justice around the treatment of people with mental illness/disorder on what they ‘know’ or are perceived to know by others but also potentially unwise to limit justice to the epistemic domain.

### 3.3 Incorporating Epistemic Failures and Lack as Part of Distress

The significance of changing perceptions and sensory information during mental distress experiences, and the epistemic instability it can produce, needs elaboration to further demonstrate the problems of this issue for lived experience research. In an article on troubling lived experience, Grant (2014) points out the ‘developing and increasing accrual of experience of this self is equally assumed to provide a stable and reliable source of knowledge and knowing’ (546). In contrast to this assumption, we know that distress experiences can destabilise one’s knowledge of themselves (Fusar‐Poli et al. 2023, 2022) and it cannot be assumed that self‐understanding is straightforwardly progressive during or after a period of mental ill‐health.

Feelings of (epistemic) failure and not knowing can be painful experiences that are a part of the overall distress experienced. The side‐lining of people with mental illness/disorder often stems from the idea that their thinking or reasoning is flawed in a significant way. Their competency as a knower is arguably contested and can be somewhat negated by their diagnostic label (e.g., psychosis or personality disorder). They can be called to describe how their knowing or reasoning actually works to a socially‐sanctioned professional. A professional may then make a judgement about their mind/brain, and whether their knowledge of the world (epistemic) or of reality (ontological) is flawed/insufficient/different. Borrowing from Dular and Ward's work (2024) on internalised oppression, I would argue that people with mental illness are sometimes assessed as ‘incompetent failed knowers’ (8).

Being considered a failed knower represents a significant issue for epistemic injustice in mental health given the origins of the concept. M. Fricker (2007) built on previous work related to the epistemology of testimony by Elizabeth Fricker and colleagues (E. Fricker 1994, 1995; E. Fricker and Cooper 1987). Elizabeth Fricker describes how knowledge is gained via competent language use such as individual testimony (1994). In her work, the focus is on the informant being ‘reliable’ and ‘trustworthy’ (E. Fricker 2006, 600) for successful knowledge transmission. Furthermore, trustworthiness can be split into speaker ‘sincerity’ and ‘competence’ (600). However, people exhibiting signs of psychosis are generally not considered in many societies to be good informants about reality because of experiences such as delusions (Gilleen and David 2005). This highlights some problems with straightforward applications of epistemic injustice arguments to lived experiencers.

It might be argued that someone experiencing psychosis could be said to show what E. Fricker (1994) considers to be an ‘honestly expressed false belief’ (150) although her work does not attempt to explore mental ill‐health. For E. Fricker (1994), the way for people to avoid drawing on this false source of epistemic knowledge is by ‘an informed assessment of a speaker's competence about some subject will typically require that the hearer already know something of the speaker's cognitive talents and failings.’ (150). This focus on assessing the competence, talents and failings of a speaker (e.g., lived experiencer) demonstrates more clearly that the stream of work underpinning how individual testimony contributes to knowledge runs into conceptual problems in the context of mental illness/disorder. Resultingly, there is a pressing need to extend social and individual understandings of the ontological domain, to develop greater respect and value of the ontological differences between people which are not necessarily centred on knowing and understanding.

We might also consider the value of conducting deeper analysis on ontological differences because they also represent something social. Take for example the long history of overdiagnosis and misdiagnosis of schizophrenia in people from minoritised Black backgrounds reported in the psychiatric research literature (Jegarl et al. 2023). Firstly, this is social phenomenon of psychiatrists’ discrimination and bias towards Black people that should be investigated with respect to it operating dyadically, rather than focusing on the lived experience of one party, which seems incomplete. Additionally, having been wrongly diagnosed within the psy paradigm, a Black individual experiencing further racism from the professional (designated to help them) engenders an ontologically different experience compared to people not exposed to racist professional encounters. The experience likely adds to confusion or disbelief. Logically the two realities (between the patient and professional) conflict and indicate how the psychiatric profession could worsen one’s mental state by undermining their sense of reality for a particular racial group. Growing paranoia can have a basis in reality for racialised minorities and, at times, may have been protective rather than pathological such as in this case where black people are more likely to be misdiagnosed.

Deleuze and Guattari (1983) have argued that unusual experiences, including those seemingly hard to understand at an individual level, can carry important conceptual value useful for social analysis. In a multi‐method umbrella review, incorporating meta‐analyses and interviews, Lazaridou et al. (2023) develop a complex and comprehensive picture of racism and psychosis for Black and racially minoritised people. The authors measured the incidences of different manifestations of psychosis but also used interviews of lived experiencers to explore how racism lays the foundations for psychosis experiences by creating destabilising conditions for self‐questioning and a sense of differentness through one’s psyche and sense of reality. As such, exploring the ontological domain in greater depth and through theorising about it can improve our collective understanding of distress phenomena.

### 3.4 The Assumption of Epistemic Goodness and Knowledge Gathering

The use of ‘knowing’ as an anchor point for mental health lived experience has been argued in this article to be problematic at times. It is important to recognise that there are types and forms of knowing in all societies in which the dominant cultures crowds out or silences other forms and representations of experience. In Anglo‐American contexts, the focus on credibility, accuracy, and trustworthiness in testimony (e.g., E. Fricker and Cooper 1987; E. Fricker 2006) appears to be driving a science of lived experience in line with these dominant cultural beliefs. Lorraine Code (2004) describes the obsession with knowledge as ‘epistemophilic societies (= societies in love with knowledge)’ (299). Townley (2006) expands that knowledge ‘is not only presumed good, even good for humanity, but presumed to be always good, and the only epistemic good. However, treating other epistemic agents purely as instruments for obtaining knowledge conflicts with the commitments required to maintain a trustful relationship’ (42). The present article brings to light questions about whether all knowledge gathering is good. As outlined earlier, people in serious distress might be evaluated as being not knowers, faulty or failed knowers of reality in the course of mental health assessment. Therefore, using them as instruments for obtaining knowledge for research purposes seems theoretically confused.

As mentioned earlier, racialised lived experiencers can face oppression in knowledge gathering research processes, just as in wider society, and tend to be underrepresented in research (Kalathil 2013). The traditional social powers used to assess the credibility of an individual’s account and the value of their contribution still operate in lived experience research and practice, albeit through institutional and organisational processes (Voronka 2016). Taking this further, Voronka (2016) asserts that ‘we need to mark how ‘people with lived experience’ risks reproducing and replicating systems of domination that privileges subjects and subjectivities that fit closely within the White heteropatriarchal matrix of ruling, which in turn risks reproducing representation that aligns with White, middle‐class, sanist, capitalist and biomedical investments’ (197). These assertions throw doubt on unquestioned assumptions that knowledge gathering of lived experience in mental health is only good.

### 3.5 Responsibility and Blame for One’s Distress Experiences

Worryingly, the rise in responsibilisation (Foucault 1977, 1988; N. Rose 1999) frames individuals as the engines of control over their own health and choices, and moves away from social, community and central governmental power. This increasingly locates individuals as responsible for their own health, mental health and their outcomes in life (see N. Rose 1999). Individual responsibility for one’s mental distress experiences and the associated self‐blame/self‐hatred can contribute to further marginalisation and, paradoxically, mental health service avoidance (Aves 2024).

Some aspects of unusual experiences may not easily link to mistreatment by others *at the point of distress* because of the unpredictable temporality of trauma and abuse which likely differs from person to person. An example of this is the long‐term and ongoing effects of having experiencing childhood abuse many years later (Feiring and Taska 2005). Lived experiencers can suffer shame and self‐blame when childhood abuse develops into abusive behaviour or self‐injury later on, for which they are told they are responsible (as an adult) (Coffey et al. 1996) which generally might be an extension of the responsibilisation agenda. In this regard, sharing details about stigmatising events for lived experience research comes with potential risks of further harm to the experiencer through blame and self‐blame (Aves 2024). This likely places particular groups at higher risk of harm during knowledge gathering processes (e.g., trauma and abuse survivors) which troubles the assumed goodness of epistemic endeavours in mental health and these concerns should be considered more widely in research.

## 4 Ontological Implications and Directions

Having questioned some assumptions about epistemic justice and argued for the overlooked importance of the ontological domain to the mental health research field, I now turn to the significance of these issues to a lived experiencer. Does collective underdevelopment of ontological framing and theorising affect individuals in their daily lives? I would argue it does. For example, in a recent study of repeated microaggressions by psychiatrists towards people who experience psychosis, findings outlined that as well as ignoring one’s account, microaggressions were experienced as dehumanising (with existential consequences). For example, ‘I often dissociated with him because I found it so horrible. I remember that I just stepped out of my body and saw myself sitting on that couch. “Joyce”’ (Defourny et al. 2024, 4). In this example, Joyce being ignored by her psychiatrist had harmful ontological consequences in the moment. For someone in serious mental distress further dehumanisation might extend the use of their working coping mechanisms of separating from their body through dissociation which is a shift in one’s sense of reality. However, research findings are typically framed in the epistemic terms of being silenced and ignored as a knower, which limits ontological theory development because we commonly overlook the ontological frame.

More than 60 years ago in the context of schizophrenia, R. D. Laing developed the notions of ‘ontological security’ and ‘ontological insecurity’ relating to the extent one experiences themself as ‘real, alive, whole, and, in a temporal sense, a continuous person’ (Laing 1964, 39). In a recent return to phenomenological psychiatry, this term has been revived exploring the ontological domain for other lived experiencers of mental distress (Spencer and Kidd 2023). This work focuses on how ontological differences raise (hermeneutical) problems for shared meaning‐making and knowledge in co‐production and professional encounters and as such is still closely tied to epistemic goals (Spencer and Kidd 2023). Presently, phenomenological psychiatry research can often focus on how to integrate these types of experiential material and understandings into applied clinical practice and tends to be framed within epistemic injustice terms (Ritunnano et al. 2023). However, Humpston (2022) critiques the epistemic authority that refers to false, unreal or delusional experiences in the context of schizophrenia. She argues that this approach does not attempt to understand the schizophrenic experience which can include paradoxes, incomprehensibility, seemingly ‘ontologically impossible’ (5) experiences which can be painful to endure but carry significant meaning. Humpston (2022) calls for deeper ontological examination to reckon with the complexity of experiences in schizophrenia and go beyond the surface of them not appearing coherent or making sense.

Research into the ontological domain in particular forms of mental distress adds further dimensions which will likely differ across people who are categorised in particular ways compared to others. I will use the example of emotionally unstable personality disorder (EUPD/BPD) for which I have been given a diagnosis for the last two decades. I have had considerable contact with mental health services in relation to this diagnosis including 5 years of going in and out of in‐patient psychiatric care and, presently, I receive community‐based care. Ontological considerations require thinking through how our first‐person realities differ (and epistemically, we can develop a shared understanding). I tend to have rapidly shifting and intense emotions, but it is a feature that roots very closely and tracks my experience of reality often very appropriately for me. I do not agree that it represents a diminishment per se (e.g., unstable or disorder) but my sense of reality can be particularly confusing under stress and so it likely has interesting ontological features. After 20 years with a diagnosis and with treatment, I still have a very limited idea of the sense of reality that other people are considered to have, described as a ‘stable’ sense of self and emotions, in contrast to my perceived instability. How is their stability characterised and embodied over time? How does someone *without* the diagnosis experience reality differently to me, for example? Research informing others about the truth of my experiences or my epistemic contribution, although validating, may not help *me* understand others better or bridge ontological differences that arise.

People with a diagnosis of EUPD/BPD might have to a dynamic way of thinking (Lewis 2023), akin to a dynamic subjectivity which might be different but is no less real than other people. For example, holding and oscillating quickly between incompatible beliefs could be ontologically valuable as has been argued in the case of people diagnosed with schizophrenia (Humpston 2022). The lived experiences of people who might attract a diagnosis of EUPD/BPD might engender epistemic pluralism which is needed to envelop a dynamic subjectivity as suggested by Lewis (2023). We could research how different ontological experiences link to the epistemic domain in line with this. I would suggest that the current article has been formulated using my propensity for epistemic pluralism because I am attempting to conceptualise across incompatible paradigms. I use this lived experience example to demonstrate how an ontological difference of dynamic subjectivity lends itself to different epistemic assumptions about the nature of truth, accuracy and credibility (Lewis 2023) and how it presents opportunities, not only for people who are living in a different way but also in research.

In mental health research, we should be calling the ontological into the frame as an area of inquiry to open up the grounds of possibility relating to assumptions and representations of reality. This focus would not be lived experiencers’ knowing capacity or understanding, and would not require formulating knowledge or a story/narrative but how they prefer to represent or speak about reality. Epistemically, we will theorise about this (it is research work), but the subject of our exploration is the ontological domain. For example, we might consider expanding the way we understand how, or why, creative endeavours or therapies are helpful in terms of whether they meet ontological needs. Might it be that some allow *expression* of one’s sense of reality (thought art, music, dance, sounds or visuals) without necessarily having to produce knowledge and understanding of oneself to demonstrate one’s value? For example, in De Witte et al.’s (2021) review on mechanisms of change in 67 studies of creative arts therapies, the authors found a number of factors relating to reality rather than knowledge, such as agency, embodiment, connection with oneself and a sense of modulating time and space. The supportive aspects of representing oneself creatively, particularly when one’s knowledge system or beliefs might be different or confusing to others, might map onto notions of ‘recognition respect’ (38) which is a concept postulated in Stephen Darwall’s work, ‘Two kinds of respect’ (1977). Recognition respect is the regard we might consider is owed to all people. The other type, appraisal respect, might be offered after some judgements or evaluations are made along criteria deemed significant and this tends to be merit‐based (e.g., judgements about credible knowledge might engender appraisal respect).

The importance of recognition in distress experiences has also been outlined in Rashed’s (2019) work on how Mad activism is tied to the demand for recognition. Furthermore, Oliver’s (2001, 2015) concept of ‘witnessing’ goes beyond recognition and could be important to lived experiencers. Witnessing is about the value of bearing witness to the unseen or unknowable, and Oliver (2015) is acknowledging that some experiences are outside what we can recognise (she uses the example of accounts of slavery and genocide to demonstrate the limits of recognition of particular experiences). In such cases, we cannot always recognise but we can witness another person’s subjectivity and vulnerabilities through offering our attention and presence in response. Oliver (2015) argues that a particular focus on truth or factual accuracy can sometimes undermine intersubjective relations because severe trauma can impact memory and perception, and marginalised positionality can impact one's capacity to build a credible and factual account through processes of exclusion or abuse. These concepts of recognition respect and witnessing could represent avenues that researchers could explore to support lived experiencers better and allow them to express themselves in ways that relate to the ontological domain without focusing on knowledge, truth and understanding. This might be particularly important to those deemed not to know or understand or those deemed to lack capacity during their distress experiences. Ontological exploration could augment and develop social justice in mental health lived experience research, in conjunction to the diverse and important work that continues to pursue epistemic justice goals (e.g., Kidd and Carel 2019; Kidd et al. 2023).

## 5 Discussion and Directions

The current focus on epistemic contributions is limiting because mental health researchers could be contributing to a growing ontological and epistemic chasm relating to the representation/expression of one's sense of reality and their knowledge. This misses explorations of the needs of lived experiencers that are not knowledge‐related. For example, respect is not necessarily only centred around knowing; a person can be respected because all beings are worthy of regard (Darwall 1977). Sweeney (2016) also outlines that respect and empowerment are key goals for Mad studies research and for survivors/activists. Even though Frantz Fanon has been noted as an inspiration for the development of Mad studies movements (Beresford and Rose 2023), he made specific reference to the ontological domain and this is still underexplored presently, particularly for racialised lived experiencers (as I am). In this article, I have outlined how we might frame this exploration in ontological terms, rather than epistemic, in line with Fanon ([1967] 2008). For racialised and marginalised lived experiencers, we should attempt to find ways to respectfully explore different senses of reality, acknowledging there are powerful social location differences between, for example, White researchers and racialised research participants. It is after developing trust and safety for individuals to represent or express their sense of reality that researchers can work towards sense‐making and understanding in line with epistemic goals. Researchers might want to explore whether processes related to achieving recognition respect and bearing witnessing can provide safer ground for sharing experiences and perhaps might be important therapeutic factors in mental health practice settings.

Research into distress experiences should be flexible and inclusive enough to incorporate the dynamic way lived experiencers represent and understand reality, particularly across time, to capture how this can and does change. Mental distress can alter one's ontological position and sense of reality because it can involve changing perceptual and sensory information and signals (Fusar‐Poli et al. 2022). Distress experiences can include a loss of safety and security in one's own body (Fusar‐Poli et al. 2022). In the context of the lived experience of depression, Fusar‐Poli et al. (2023) conclude that the ‘existential shift in how one finds oneself in the world can be expressed not only in terms of emotions, body, self or time’ (362) but also in the structure of experience. This signals the opportunity to explore ontological features over time to better understand existential shifts for experiences such as feeling depressed and other distress experiences. Researching these changes and reflections by lived experiencers can help us better understand (epistemic goals) but also support lived experiencers to represent or portray their different senses of reality (ontological goals).

Longitudinal work on lived experiencers’ changing reflections could support the development of methodological frameworks that are needed which allow for one’s representation of reality and their sense‐making to change over the life course. This direction acknowledges the interactive temporal nature of lived experience described at the outset of this work (Sadala and Adorno 2002). Presently, accounts and testimony of lived experience tends to be rooted in cross‐sectional reflections over the recent or distant past (e.g., Punton et al. 2022). Bury’s classic idea of ‘chronic illness as biological disruption’ (1982) offers significant potential for better conceptualising lived experience in mental health by framing it with respect to the ongoing temporal dimension and is in direct contrast with the view that illness is a largely stable identity. Bury (1982) writes about the insidious onset of chronic illness: ‘Non‐communicable diseases do not “break‐out” they “creep‐up”’ (170). As such exploring within the ontological frame (of one’s sense of reality) over a longer‐term journey might invite complex and changing interpretations of earlier experiences and different self‐understandings later on which brings much experiential material into scope for collective (and self) understanding.

## 6 Conclusions

Situating mental health lived experience so heavily within the epistemic domain comes with unresolved problems and tensions. The focus on epistemic knowledge and the trustworthiness and credibility of one’s account is at odds with the confusing experiences often associated with many forms of mental distress. Furthermore, the idea of one’s account or testimony implies a sort of coherence and lack of confusing positions which may not be the case for the lived experiencers, such as myself, who often hold (and live with) impossible or contradictory beliefs. In such cases, accessing one’s own perspectives and experiences may be phenomenologically very complex.

In mental health, lived experiencers’ knowledge can be questioned on account that their sense of reality differs from others which is an ontological difference. It should be more widely recognised that mistreatment towards lived experiencers can also emanate from the ontological domain, in addition to the epistemic domain (which is well accepted), because of social judgements about their mind/brain. Future research could aim to explore how people can *represent* and *portray* their sense of reality (to foster respect for their ontological differences), in addition to how they *explain* it for collective understanding and sense‐making (to foster knowledge and understanding). It will be important not to replicate or promote existing power structures around whose sense of reality is superior such as by over‐relying on categories or psychiatric diagnostic labels. Across many psy disciplines and even some Mad studies movements, there is a need to widen the scope of lived experience research towards deeper examination by exploring the link between one's sense of reality and their knowledge in order to bridge the ontological and epistemic domains.

## Author Contributions

**Rajvinder Samra:** conceptualization (lead), formal analysis (lead), investigation (lead), methodology (lead), writing – original draft (lead), writing – review and editing (lead).

## Ethics Statement

The author has nothing to report.

## Conflicts of Interest

The author declares no conflicts of interest.

## Data Availability

Data availability is not applicable to this article as no new data were created or analysed in this article.
